# Differential Impact of Low Voltage Area Radiofrequency Ablation on Recurrence of Atrial Fibrillation Versus Atrial Tachycardia in Patients With Persistent Atrial Fibrillation

**DOI:** 10.1161/CIRCEP.125.014408

**Published:** 2026-04-01

**Authors:** Daisuke Sakamoto, Akihiro Sunaga, Yuki Matsuoka, Hideaki Hasegawa, Hirota Kida, Tomoharu Dohi, Daisaku Nakatani, Katsuki Okada, Tetsuhisa Kitamura, Masaharu Masuda, Nobuaki Tanaka, Tetsuya Watanabe, Hitoshi Minamiguchi, Yasuyuki Egami, Takafumi Oka, Tomoko Minamisaka, Takashi Kanda, Masato Okada, Masato Kawasaki, Yasuhiro Matsuda, Koji Tanaka, Nobuhiko Makino, Shungo Hikoso, Koichi Inoue, Yohei Sotomi, Yasushi Sakata, and the OCVC-SUPPRESS-AF Investigators, Masaharu Masuda

**Affiliations:** Kansai Rosai Hospital, Amagasaki, Japan; Kansai Rosai Hospital, Amagasaki, Japan; Kansai Rosai Hospital, Amagasaki, Japan; Kansai Rosai Hospital, Amagasaki, Japan; Kansai Rosai Hospital, Amagasaki, Japan; National Hospital Organization Osaka National Hospital, Osaka, Japan; National Hospital Organization Osaka National Hospital, Osaka, Japan; National Hospital Organization Osaka National Hospital, Osaka, Japan; National Hospital Organization Osaka National Hospital, Osaka, Japan; National Hospital Organization Osaka National Hospital, Osaka, Japan; Osaka General Medical Center, Osaka, Japan; Osaka General Medical Center, Osaka, Japan; Osaka General Medical Center, Osaka, Japan; Osaka General Medical Center, Osaka, Japan; Osaka General Medical Center, Osaka, Japan; Osaka Keisatsu Hospital, Osaka, Japan; Osaka Keisatsu Hospital, Osaka, Japan; Osaka Keisatsu Hospital, Osaka, Japan; Osaka Keisatsu Hospital, Osaka, Japan; Osaka Rosai Hospital, Sakai, Japan; Osaka Rosai Hospital, Sakai, Japan; Osaka Rosai Hospital, Sakai, Japan; Osaka Rosai Hospital, Sakai, Japan; Osaka University Graduate School of Medicine, Suita, Japan; Osaka University Graduate School of Medicine, Suita, Japan; Osaka University Graduate School of Medicine, Suita, Japan; Osaka University Graduate School of Medicine, Suita, Japan; Osaka University Graduate School of Medicine, Suita, Japan; Osaka University Graduate School of Medicine, Suita, Japan; Osaka University Graduate School of Medicine, Suita, Japan; Osaka University Graduate School of Medicine, Suita, Japan; Osaka University Graduate School of Medicine, Suita, Japan; Osaka University Graduate School of Medicine, Suita, Japan; Osaka University Graduate School of Medicine, Suita, Japan; Osaka University Graduate School of Medicine, Suita, Japan; Osaka University Graduate School of Medicine, Suita, Japan; Osaka University Graduate School of Medicine, Suita, Japan; Osaka University Graduate School of Medicine, Suita, Japan; Osaka University Graduate School of Medicine, Suita, Japan; Osaka University Graduate School of Medicine, Suita, Japan; Osaka University Graduate School of Medicine, Suita, Japan; Sakurabashi Watanabe Hospital, Osaka, Japan; Sakurabashi Watanabe Hospital, Osaka, Japan; Sakurabashi Watanabe Hospital, Osaka, Japan; Sakurabashi Watanabe Hospital, Osaka, Japan; Sakurabashi Watanabe Hospital, Osaka, Japan; Yao Municipal Hospital, Yao, Japan; Yao Municipal Hospital, Yao, Japan; 1 Department of Cardiovascular Medicine, The University of Osaka Graduate School of Medicine, Suita (D.S., A.S., Y. Matsuoka, H.H., H.K., T.D., D.N., K.O., T.O., Y. Sotomi, Y. Sakata).; 2 Department of Medical Informatics, The Univ of Osaka Graduate School of Medicine (K.O.); 3 Department of Social and Environmental Medicine, The Univ of Osaka Graduate School of Medicine (T. Kitamura).; 4 Cardiovascular Center, Kansai Rosai Hospital, Amagasaki (M.M., Y. Matsuda).; 5 Cardiovascular Center, Sakurabashi Watanabe Advanced Healthcare Hospital, Osaka (N.T., M.O., K.T.).; 6 Department of Cardiovascular Medicine, Yao Municipal Hospital (T.W., T.M.).; 7 Cardiovascular Division, Osaka Keisatsu Hospital (H.M., T. Kanda, N.M.).; 8 Division of Cardiology, Osaka Rosai Hospital, Sakai (Y.E.).; 9 Division of Cardiology, Osaka General Medical Center (M.K.).; 10 Cardiovascular Medicine, Nara Medical University, Kashihara (S.H.).; 11 Cardiovascular Division, National Hospital Organization Osaka National Hospital, Japan (K.I.).

**Keywords:** atrial fibrillation, catheter ablation, informed consent, pulmonary vein, tachycardia

To evaluate the efficacy of low-voltage area (LVA) ablation combined with pulmonary vein isolation (PVI) for persistent atrial fibrillation (AF), we conducted the SUPPRESS-AF trial and reported a trend toward lower 1-year recurrence of AF or atrial tachycardia (AT) by adding LVA ablation to PVI in patients with persistent AF and coexisting LVAs.^1^ However, the original trial evaluated the recurrence of both AF and AT together. Some postablation AT recurrences have been reported to originate from reentrant substrates, such as electrical scars and slow-conduction channels created by extensive lesion sets.^2,3^ Therefore, LVA ablation could increase the risk of iatrogenic AT recurrence, although it may reduce the AF substrate and consequently decrease AF recurrence. A balanced assessment of these conflicting effects has yet to be established. In this study, using the data from the SUPPRESS-AF trial, we separately evaluated the effect of LVA ablation on AF and AT recurrence.

A multicenter, randomized, open-label SUPPRESS-AF trial was conducted at 8 centers in Japan. The trial was approved by the ethics committee of the University of Osaka and each participating hospital, and all participants provided written informed consent. The trial enrolled patients undergoing initial radiofrequency catheter ablation for persistent AF with left atrial LVAs (defined as areas with a bipolar peak-to-peak voltage <0.50 mV) covering ≥5 cm^2^ of the left atrial surface on voltage map after PVI. Participants were randomly assigned in a 1:1 ratio to undergo LVA ablation (PVI+LVA-ABL group) or not (PVI-alone group). Details of the study protocol were previously published.^1^ The recurrence of AF or AT was monitored using ECG, including 24-hour Holter ECG at 6 and 12 months, and twice-daily and symptom-driven 30-second recordings with a portable ECG from 6 to 12 months. We employed a Fine–Gray model to evaluate the risk of AF recurrence, considering AT recurrence and death as competing risks. Similarly, the risk of AT recurrence was assessed with AF recurrence and death treated as competing risks. The data that support the findings are available on reasonable request.

Among 1347 consecutive patients with PVI followed by voltage mapping (June 2019–August 2022), LVAs were identified in 343 (25.5%). After excluding one patient due to an allocation error, 342 were randomized (170 PVI+LVA-ABL group versus 172 PVI-alone group). One patient in the PVI-alone group was subsequently excluded due to an eligibility violation, resulting in 341 patients for the end point assessment. During the 1-year observation period, 39 patients experienced AF recurrence, 22 experienced AT recurrence, and 2 died in the PVI+LVA-ABL group. In the PVI-alone group, 58 patients had AF recurrence, 13 had AT recurrence, and 1 died. Compared with the PVI-alone group, the PVI+LVA-ABL group had a significantly lower cumulative incidence of AF recurrence (23% versus 34%, subdistribution hazard ratio, 0.63 [95% CI, 0.42–0.94]; *P*=0.023). The incidence of AT recurrence was numerically higher in the PVI+LVA-ABL group, although this difference did not reach statistical significance (13% versus 8%; subdistribution hazard ratio, 1.76 [95% CI, 0.89–3.48]; *P*=0.100; Figure).

**Figure.**
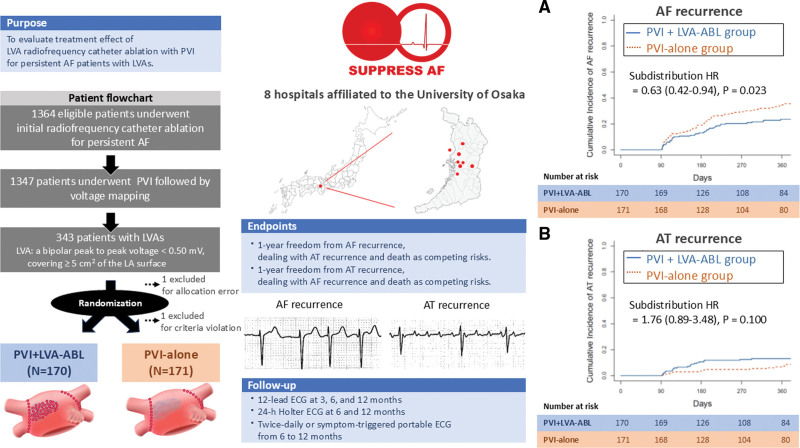
**This figure illustrates the design of the SUPPRESS-AF trial, beginning with the study purpose and patient flowchart followed by the end points and follow-up methodology.** It also presents the cumulative incidence curves for atrial fibrillation (AF) recurrence (**A**) and atrial tachycardia (AT) recurrence (**B**). ABL indicates ablation; HR, hazard ratio; LA, left atrium; LVA, low voltage area; and PVI, pulmonary vein isolation.

LVA ablation significantly reduced AF recurrence after accounting for competing risks of AT recurrence and death. In persistent AF patients with LVAs, in which both advanced left atrial electrophysiological and structural remodeling are presumed, suppression of focal triggers by PVI alone may be insufficient, and additional substrate modification through LVA ablation may be crucial for durable AF control.^4,5^ Conversely, the numerically higher—albeit not statistically significant—rate of AT recurrence following LVA ablation supports the hypothesis that postablation AT recurrences are predominantly iatrogenic, arising from slow-conduction channels or reentrant circuits created by insufficient ablation lesions.^2^

The limitation of the study lies in the fact that only the first event to occur, either AF or AT, was evaluated. This approach did not account for the recurrence of AF after AT recurrence, or the recurrence of AT following AF recurrence. In addition, we did not collect quantitative arrhythmia burden nor systematically capture rate-control adjustments or symptom metrics, precluding evaluation of cumulative AF/AT load and its long-term clinical relevance. Furthermore, both AF and AT recurrences were assessed by 24-hour Holter ECG and portable ECG monitoring, without intracardiac electrogram data, which might have resulted in an underreporting of recurrence. Because the study was conducted in a single country, a racially homogeneous population in Japan, both the trial steering committee and the participant population lacked demographic diversity. In addition, sex-specific outcomes were not analyzed or reported. These factors may limit the generalizability of findings and the interpretation of potential subgroup differences.

In conclusion, LVA ablation combined with PVI significantly reduced AF recurrence compared with PVI alone, while the incidence of AT recurrence was numerically higher. The data from the second ablation during the follow-up in the SUPPRESS-AF trial, incorporating invasive mapping data, may provide insights into the underlying mechanisms of AT recurrence and clarify whether AT recurrence links to sites of LVA ablation.

## Article Information

### Acknowledgments

The authors thank the clinical engineers Takashi Sumigawa, Naoya Kurata, Yusuke Ikada, Yoshitaka Kikuchi, Atsushi Shiono, and Hiroshi Kobayashi for their dedication in protocol creation and operation of ablation-related equipment according to the protocol; and the clinical research coordinators Nagisa Yoshioka, Satomi Kishimoto, Kyoko Tatsumi, and Yumi Yoshida for their excellent assistance in data collection, data management, and secretarial administration.

### Disclosures

Dr Inoue received honoraria from Johnson & Johnson, Medtronic, Boston Scientific, and Daiichi Sankyo, and a study grant from Johnson & Johnson. The other authors report no conflicts.

## Appendix

The OCVC-SUPPRESS-AF InvestigatorsKansai Rosai Hospital, Amagasaki, Japan: Masaharu Masuda, Takashi Kanda, Yasuhiro Matsuda, Hiroyuki Uematsu, Toshiaki Mano. National Hospital Organization Osaka National Hospital, Osaka, Japan: Koichi Inoue, Tsuyoshi Mishima, Tatsuhisa Ozaki, Takuya Ohashi, Yasunori Ueda. Osaka General Medical Center, Osaka, Japan: Tetsuya Watanabe, Yoshio Furukawa, Masato Kawasaki, Mitsutoshi Asai, Takahisa Yamada. Osaka Keisatsu Hospital, Osaka, Japan: Nobuhiko Makino, Hitoshi Minamiguchi, Akio Hirata, Yoshiharu Higuchi. Osaka Rosai Hospital, Sakai, Japan: Yasuyuki Egami, Masamichi Yano, Yasuharu Matsunaga-Lee, Masami Nishino. Osaka University Graduate School of Medicine, Suita, Japan: Yasushi Sakata, Shungo Hikoso, Daisaku Nakatani, Hiroya Mizuno, Toshihiro Takeda, Takafumi Oka, Tomoaki Nakano, Kentaro Ozu, Takayuki Sekihara, Katsuki Okada, Tomoharu Dohi, Yohei Sotomi, Akihiro Sunaga, Hirota Kida, Bolrathanak Oeun, Taiki Sato, Yuki Matsuoka, Daisuke Sakamoto. Sakurabashi Watanabe Hospital, Osaka, Japan: Nobuaki Tanaka, Masato Okada, Koji Tanaka, Yuko Hirao, Katsuomi Iwakura. Yao Municipal Hospital, Yao, Japan: Tomoko Minamisaka, Shiro Hoshida.
